# Defending Against State-Inducing Spoofing Attacks in Intelligent Connected Vehicles: A Real-Time Temporal Feature Fusion Framework

**DOI:** 10.3390/s26154758

**Published:** 2026-07-27

**Authors:** Chen Dong, Hao Wu, Cheng Li

**Affiliations:** School of Electronic and Information Engineering, Beijing Jiaotong University (BJTU), Beijing 100044, China; 19111011@bjtu.edu.cn (C.D.); chengli@bjtu.edu.cn (C.L.)

**Keywords:** CAN bus, intrusion detection, hybrid attention mechanism, intelligent connected vehicles, advanced driver assistance systems, in-vehicle network security

## Abstract

As intelligent connected vehicles (ICVs) integrate advanced driver-assistance systems (ADAS) and autonomous-driving functions, CAN bus attacks have become more diverse in mechanism and safety impact. Beyond flooding or direct command injection, state-inducing spoofing attacks inject falsified CAN frames to manipulate vehicle-state signals. Rather than directly controlling vehicle behavior, they mislead ADAS state estimation, potentially triggering inappropriate control responses and threatening driving safety. Existing intrusion detection methods mainly target conventional CAN attacks and limited operating states, leaving limited detection generalization in complex attack scenarios. Accordingly, this paper proposes MTFF, a multi-scale temporal feature fusion framework for CAN intrusion detection. MTFF builds two complementary CAN streams: an intra-ID kinematic sequence capturing short-term state continuity under the same identifier and an inter-ID scheduling sequence capturing timing relationships among neighboring frames, thereby characterizing CAN traffic from state-continuity and scheduling-relation perspectives. Multi-scale 1-D convolutions extract local temporal features, while positional self-attention and symmetric cross-attention model long-range dependencies and fuse the streams to detect contextual temporal and state inconsistencies. Experiments on multi-vehicle CAN datasets covering representative operating states show that, in the most challenging setting, MTFF achieves F1-scores above 0.94 on two production vehicles, with per-frame latency below 0.006 ms.

## 1. Introduction

Intelligent connected vehicles (ICVs) are rapidly integrating vehicle-to-everything (V2X) connectivity and increasingly sophisticated autonomous-driving functions, which has led to an increase in both the number and functional diversity of onboard electronic control units (ECUs) [1]. Meanwhile, vehicle electronic control units (ECUs) are rapidly evolving from isolated embedded controllers into highly coordinated computing nodes that support safety-critical chassis, steering, braking, and powertrain functions. Recent research on robust game-theory control for all-wheel steering further shows that advanced ECU-supported control strategies are being developed to enhance vehicle handling stability under complex driving conditions [2]. This trend indicates that modern vehicle functions increasingly depend on timely and trustworthy in-vehicle data exchange. Recent industry reports indicate that the penetration of Advanced Driver Assistance Systems (ADAS) continues to rise in the connected-vehicle market [3,4]. As a result, the reliability and security of in-vehicle communication have become increasingly important to both functional safety and user trust.

Moreover, the development of ICVs is increasingly moving from single-vehicle intelligence toward cooperative vehicle–infrastructure–cloud systems. In this paradigm, vehicles, roadside infrastructure, edge nodes, and cloud platforms cooperate through V2X communication to support cooperative perception, vehicle localization, state estimation, and driving decision-making. Research on RIS-assisted vehicle localization with unknown inputs further shows that such collaborative systems must not only improve localization and state-estimation robustness under uncertain traffic environments and complex propagation conditions, but also protect vehicle-location and signal privacy through lightweight mechanisms such as output-mask strategies [5]. This broader trend indicates that the security of ICVs has extended beyond traditional in-vehicle communication protection toward broader system-level issues, including cross-domain state fusion, collaborative computation, and privacy preservation.

Within this broader system-level security context, the Controller Area Network (CAN) bus remains the primary communication backbone for chassis and powertrain domains, carrying critical sensing and control frames that support ADAS functions. However, the legacy CAN protocol lacks native encryption and source authentication, leaving the in-vehicle network vulnerable to malicious injections. Existing studies have shown that attackers can exploit this weakness to launch conventional CAN-bus attacks, such as flooding and replay attacks, thereby disrupting network availability and communication stability. In a landmark 2015 study, Miller and Valasek remotely compromised a Jeep Cherokee and manipulated its acceleration, braking, and steering through malicious CAN traffic injection [6]. More importantly, this vulnerable CAN bus can also serve as an attack medium for state-inducing spoofing attacks against ADAS, creating a more covert and critically important security threat. By injecting falsified CAN frames carrying manipulated vehicle-state signals, an attacker can distort the state estimation of ADAS functions that rely on these frames for control. This may further induce erroneous control responses and threaten driver and passenger safety [7]. For example, spoofed yaw-rate frames may cause the lane-keeping controller to infer a nonexistent trajectory deviation and issue an erroneous steering correction.

To protect CAN networks, researchers have proposed a variety of defense mechanisms, including encryption and authentication [8,9], ECU access control [10], network partitioning, and intrusion detection systems (IDSs) [11,12]. Although cryptographic and access-control mechanisms are effective in principle, their deployment in production ICVs is often constrained by legacy hardware limitations and strict real-time requirements. Consequently, IDSs have become one of the most practical defense mechanisms for in-vehicle networks. Existing CAN-bus IDSs can be broadly grouped into frequency-based methods [13,14], content-based methods [15], and joint methods [16].

Despite the progress of CAN-bus intrusion detection, existing methods still exhibit two important limitations. First, most schemes are developed for conventional attacks such as denial-of-service and fuzzy attacks, and therefore rely on conspicuous anomalies in bus load, ID statistics, or payload format. Such designs are less effective against state-inducing spoofing attacks, whose injected frames can closely mimic legitimate frame structures and transmission frequencies. Second, many methods are designed and evaluated under limited operating conditions. In practice, CAN traffic characteristics vary significantly across the representative operating states of ICVs, including ignition, engine running, and steering after engine start. Ignoring these dynamic shifts often leads to degraded robustness in realistic deployments.

To address these limitations, we propose MTFF, a frame-level intrusion-detection framework based on multi-scale temporal feature fusion and hybrid attention. Unlike a single-stream representation formed by arranging mixed-ID frames in their order of arrival, MTFF employs a dual-stream temporal representation with distinct temporal scopes. Specifically, we transform raw CAN traffic into two complementary streams: an intra-ID kinematic sequence and an inter-ID scheduling sequence. The former captures the short-term continuity of vehicle dynamics, whereas the latter captures scheduling dependencies among temporally neighboring CAN frames with different identifiers (IDs) under RTOS-governed control tasks. This dual-stream construction preserves the local scheduling context while selectively retrieving same-ID frames from a longer history. It therefore avoids the substantial expansion of the mixed-ID sequence that would otherwise be required to capture sufficient same-ID history, thereby balancing historical coverage with input compactness. MTFF first employs a multi-scale 1-D CNN to extract local temporal patterns from the two streams. It then applies independent multi-head self-attention modules with positional encoding to model long-range dependencies within each stream. To further characterize the mutual dependencies between vehicle-state evolution and frame scheduling, MTFF employs a symmetric cross-attention mechanism to dynamically fuse the two streams. Through this bidirectional interaction, each stream provides contextual guidance for the other, enabling MTFF to capture subtle inconsistencies between state evolution and scheduling context that may not be apparent from either stream alone. In this way, MTFF supports accurate frame-level anomaly detection while maintaining low detection latency.

The main contributions of this paper are summarized as follows:

1. We formulate frame-level detection of state-inducing spoofing attacks in ICV CAN traffic as a compact dual-stream temporal modeling problem. By constructing an intra-ID kinematic sequence and an inter-ID scheduling sequence directly from raw CAN traffic, the proposed representation reduces input redundancy and detection latency while jointly capturing vehicle-dynamics continuity and cross-ID scheduling dependencies.
2. We propose MTFF, a frame-level intrusion-detection framework that integrates multi-scale 1-D CNN feature extraction, positional self-attention, and symmetric cross-attention fusion. This design jointly models local temporal patterns and long-range inter-stream dependencies, enabling accurate detection of four types of attacks across three representative operating states of ICVs.
3. We conduct comprehensive experiments on real-world multi-vehicle datasets under four representative attack scenarios, including conventional CAN-bus attacks and state-inducing spoofing attacks, across ignition, engine running, and steering after engine start. The results show that MTFF achieves strong detection performance with low detection latency, outperforms representative baselines, and demonstrates strong potential for real-time in-vehicle deployment.

The remainder of this paper is organized as follows. Section 2 reviews related work on intrusion detection systems for the CAN bus. Section 3 formulates the attack model and the underlying problem. Section 4 presents the proposed intrusion detection method. Section 5 describes the experimental setup and analyzes the experimental results. Finally, Section 6 concludes the paper.

## 2. Related Work

Existing CAN-bus intrusion detection methods can be broadly categorized into three groups according to the traffic cues they exploit: frequency-based methods, content-based methods, and joint methods.

(1) Frequency-based intrusion detection: Due to the presence of a time-triggered mechanism, there is a periodicity in the transmission of CAN frames that is used as an indicator for intrusion detection. In 2018, Wu et al. [17] proposed an intrusion detection method based on the information entropy of CAN messages in a sliding time window with a fixed number of messages, which has high detection accuracy for DoS and injection attacks. However, they did not evaluate the detection performance of replay and fuzzy attacks and used a single performance metric. In 2021, Han et al. [18] proposed an intrusion detection and attack identification method based on CAN ID intervals, which uses the intervals of normal traffic as the detection baseline, and then constructs machine learning-based attack identification using the multiple statistical values of the intervals as the feature space. The algorithm achieves good detection and identification performance in both vehicle data sets in four attack scenarios, but the high dependence of the detection baseline on the interval leads to the inability to cope with attacks against non-periodic frames. In 2023, Yu et al. [19] proposed an intrusion detection scheme named TCE-IDS, based on frame-based time interval conditional entropy. This approach computes the conditional entropy for each ID under normal conditions, establishing it as the baseline for detection. The algorithm was validated on the 2018 Ford EcoSport CAN network, demonstrating nearly 100% detection accuracy for DoS, fuzzy, and impersonation attacks. However, specific details regarding the attacks were not provided.
(2) Content-based intrusion detection: In 2017, Markovitz et al. [20] used the proposed greedy algorithm to classify the data domains of frames into Constant fields, Multi-value fields, and Counter or Sensor fields based on a large number of bus recordings in different scenarios. Based on this, they proposed a semantics-aware, semantics-based anomaly detection system with a relatively low false alarm rate. However, the authors did not evaluate the sensitivity of this system in real-world attack scenarios. In addition, the system is also difficult to deal with spoofing and replay attacks because they are always structurally disguised as legitimate frames. In 2020, Longari et al. [21] proposed an anomaly detection system for CAN buses based on Long Short-Term Memory (LSTM)-autoencoders called CANnolo. The authors built a model based on the data load of multiple CAN packets for each class of IDs and then detected anomalies by comparing the difference between the predicted sequence and the actual sequence. Experiments show that the algorithm has good performance in most scenarios. However, the computational cost of the time series can reach 0.95 s, which may not be sufficient for further real-time response requirements. In 2021, Tahsin et al. [22] proposed an intrusion detection system based on bus message ID sequences inspired by the work of Marchetti and Stabili [23], relying on the ID transfer matrix to detect k-frame message sequences. The experiment shows that the true positive rate for all 5 types of attacks improves to 1 when the value of k is greater than 2. However, increasing the value of k also increases detection latency as well as the difficulty of determining the attack frames.
(3) Joint intrusion detection: This method jointly exploits multiple traffic cues, such as timing, payload, and contextual dependencies. In 2021, Sun et al. [24] proposed CLAM, which combines 1-D CNN, LSTM, and a soft-attention mechanism and achieves good detection performance across five attack scenarios. However, CLAM filters 13 out of 28 identifiers when constructing the dataset and does not report detection latency, which limits its practical evaluation. In the same year, Xie et al. [25] proposed an augmented generative adversarial-network-based intrusion detection scheme that incorporates a manufacturer-provided CAN communication matrix. Based on the message block defined in [26], the authors further constructed a more fine-grained message group in which the messages share the same identifier. They also fed message types and signal ranges from the communication matrix into the GAN discriminator to improve detection accuracy. Nevertheless, this scheme relies on detailed proprietary CAN communication information, which is often unavailable in practice. Xiao et al. [27] proposed a Graph Node Attention Network (GNAT)-based intrusion detection method that constructs a topology from packet identifiers within a time window and incorporates the mean payload value as an additional feature. Although the method performs well on both the Car-Hacking and Survival Analysis datasets, its simplified treatment of the data field limits the best performance to relatively large time windows between 75 and 100. Fu et al. [28] proposed IoV-BERT-IDS, which uses BERT to extract contextual semantics from raw traffic. Although it achieves high detection accuracy and good cross-vehicle generalization, its pretraining cost is high and its suitability for real-time deployment in resource-constrained vehicular environments remains limited.

## 3. Attack Model and Problem Formulation

In contemporary ICVs, the CAN bus is exposed to a wide range of cyber threats. This section first describes the security vulnerabilities of the legacy CAN bus and explains why they are particularly critical to ADAS-enabled driving. It then summarizes conventional CAN bus attack scenarios and finally formulates the state-inducing spoofing attack considered in this paper.

### 3.1. Legacy CAN and Security Posture in ICVs

Despite the emergence of high-bandwidth protocols such as Automotive Ethernet, legacy CAN remains the primary communication backbone for powertrain and chassis domains in ICVs because of its deterministic latency, high reliability, and cost-efficiency. However, its lack of built-in encryption and source authentication leaves the CAN bus highly vulnerable to malicious CAN frame injections.

Although modern vehicle architectures attempt to mitigate these vulnerabilities through Secure Onboard Communication (SecOC), deploying such cryptographic protection universally remains impractical. Because vehicle-dynamics functions operate under stringent real-time constraints and production ECUs often rely on resource-limited Commercial Off-The-Shelf (COTS) microcontrollers, applying SecOC to high-frequency kinematic CAN frames can incur prohibitive computational overhead. As a result, critical low-level sensor frames often remain unencrypted. Modern ADAS algorithms rely heavily on these CAN frames carrying vehicle-state signals, such as wheel speeds and yaw rates, for vehicle-state estimation and trajectory planning. If an attacker tampers with such low-level sensor frames through malicious injection, the resulting deception can directly propagate into the autonomous control loop.

### 3.2. Conventional Cyberattacks on CAN Bus

**Figure 1:**
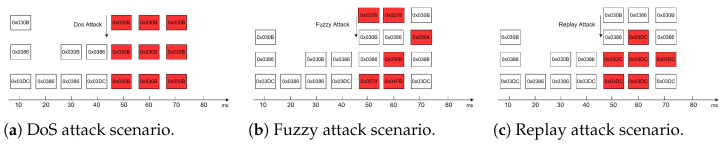
Three typical cyberattacks targeting the vehicle CAN bus.

Exploiting the broadcast nature of the CAN bus, conventional cyberattacks typically aim to disrupt network availability or communication stability. We briefly summarize three representative attacks that have been widely studied in the literature and demonstrated in real-world scenarios, as illustrated in Figure 1.

**DoS attack:** As illustrated in Figure 1a, attackers exploit the CAN bus arbitration mechanism by flooding the network with high-priority frames, typically using the smallest legitimate ID. This can induce severe network congestion or even force nodes into a bus-off state, thereby disrupting normal CAN communication and degrading vehicle functionality.

**Fuzzy attack:** As shown in Figure 1b, a fuzzy attack injects large amounts of CAN frames with highly randomized IDs and data fields to probe potential vulnerabilities in the in-vehicle network. By observing the vehicle’s responses to these anomalous frames, attackers can infer hidden system behaviors and disrupt normal communication and control processes.

**Replay attack:** As depicted in Figure 1c, attackers first eavesdrop on the CAN bus to capture legitimate communication frames and then retransmit these recorded frames without modification. When the replayed frames carry critical control-related information, such as braking or acceleration commands, they can interfere with normal vehicle behavior and pose direct risks to driver safety.

These attacks usually produce obvious macroscopic anomalies, such as sharp increases in bus load or abnormal timing patterns, and can therefore be detected relatively easily by conventional IDSs. The more difficult challenge is posed by stealthy and precise manipulations that leave no such obvious traces.

### 3.3. State-Inducing Spoofing Attack

**Figure 2:**
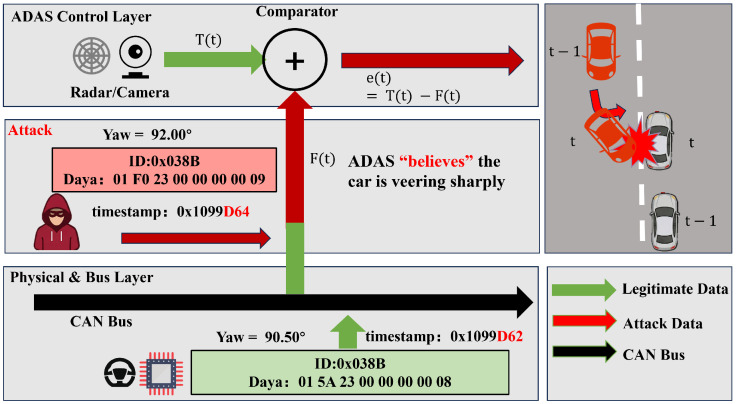
The state-inducing spoofing attack scenario, illustrating how asynchronous mailbox overwriting poisons the ADAS control-layer feedback loop and causes critical physical deviations.

We consider a more covert attack model targeting ADAS, referred to as the state-inducing spoofing attack, as illustrated in Figure 2. Unlike brute-force attacks that primarily disrupt network availability, this attack tampers with the state-feedback path by injecting falsified CAN frames carrying forged vehicle-state signals. As a result, ADAS modules process the malicious feedback F(t) instead of the legitimate target state T(t), which may ultimately lead to erroneous control actions.

This attack exploits the timing mismatch between asynchronous CAN-controller reception and periodic RTOS polling in ICV ECUs. In a typical ADAS architecture, control tasks are scheduled by the RTOS at fixed intervals, for example every 10 or 20 ms, while the CAN controller receives frames asynchronously and stores them in dedicated Receive (Rx) mailboxes according to their identifiers. Once a legitimate kinematic frame reaches the End-of-Frame (EOF) bit, it is written into the corresponding Rx mailbox. The adversary then uses the observable EOF instant of the legitimate frame on the CAN bus as an external timing reference and injects a spoofed frame with the same ID after a short delay. If the spoofed frame is written before the next RTOS polling instant, the legitimate mailbox content is overwritten, and the ADAS task reads the spoofed payload instead of the legitimate vehicle state. If the spoofed frame is delayed by bus arbitration, instantaneous bus load, scheduling jitter, or transmission delay, the overwrite attempt may fail in the current polling cycle. Therefore, this study models the state-inducing spoofing attack as a timing-constrained repeated same-ID injection attack across successive control cycles, rather than an idealized attack that necessarily succeeds in every cycle. Because this overwrite occurs at the CAN-controller Rx-mailbox level and is not explicitly visible to the upper-layer software, the attack can evade IDSs that rely only on coarse frequency or bus-load anomalies.

To implement the above attack, this study assumes that the adversary has obtained access to the in-vehicle CAN bus through a compromised in-vehicle node, a diagnostic interface, or an external communication module, and has the capability to monitor bus traffic and transmit standard CAN frames. Through bus monitoring, the adversary can observe the normal transmission period, arrival instants, and payload variation patterns of the target ID, and can transmit spoofed frames with the same identifier. However, the adversary cannot directly control the RTOS scheduler of the victim ECU, access the internal task states of the ECU, or precisely know the internal instant at which the next control task polls the Rx mailbox. Therefore, JIT injection does not require perfect synchronization with the internal ECU task clock; instead, it relies on bus-level observable events and high-resolution timed transmission.

A fundamental challenge for the adversary in ICVs is the black-box nature of proprietary CAN databases. To induce the target state without triggering format-based anomaly detection, the adversary must infer the payload structure from observed CAN traffic.

Let the data field of a target CAN frame at time step *t* be represented by a binary vector $P^{\left(t\right)}={\left[p_{1}^{\left(t\right)},p_{2}^{\left(t\right)},\ldots ,p_{L}^{\left(t\right)}\right]}^{⊤}\in {\left\{0,1\right\}}^{L}$, where *L* denotes the frame length and is typically 64 bits. By eavesdropping on the bus over an observation window, the adversary computes the information entropy H(i) of the *i*-th bit sequence to identify semantic boundaries:

(1) $$H\left(i\right)=-\sum_{v\in \{0,1\}}\mathrm{Pr}\left(p_{i}=v\right)\log_{2}\mathrm{Pr}\left(p_{i}=v\right)$$

Based on the entropy distribution, the payload index set I={1,2,…,L} is divided into a static set S={i∈I∣H(i)≈0} and a dynamic set D={i∈I∣H(i)>0}. Frames with D=∅ are treated as static requests and excluded from further analysis. For kinematic state frames, the dynamic set D is further divided into the target-state subset $D_{tar}$, which contains the bits of the manipulated state variable (e.g., yaw rate), and the correlated-dependency subset $D_{dep}$, which contains associated bits such as rolling counters or checksums.

The malicious payload $P^{*}$ is then constructed using a modulation function M and a mapping function Φ:

(2) $$p_{i}^{*}=\left\{\begin{array}{cc} p_{i}^{\left(t\right)}, & \mathrm{if}\;i\in S\\ M(p_{i}^{\left(t\right)}), & \mathrm{if}\;i\in D_{tar}\\ \Phi (P_{tar}^{*},t), & \mathrm{if}\;i\in D_{dep} \end{array}\right.$$

where $p_{i}^{\left(t\right)}$ preserves the original static bits so that the forged frame remains consistent with the legitimate ECU output. The modulation function M(·) modifies the target-state bits to the spoofed kinematic values, while the mapping function Φ(·) adjusts the verification bits in $D_{dep}$ to remain consistent with the modified target bits. In this way, the constructed payload $P^{*}$ can satisfy the relevant data-link-layer integrity constraints.

## 4. Proposed Intrusion Detection Method

**Figure 3:**
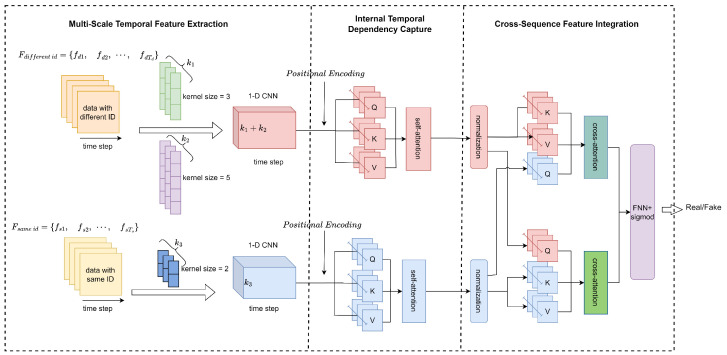
The overall framework of the proposed algorithm.

In-vehicle CAN traffic exhibits complex contextual dependencies shaped by both time-triggered and event-triggered transmission mechanisms. For example, periodic frames often show strong temporal synchronization, while kinematic payloads evolve continuously with vehicle motion. These spatio-temporal patterns are difficult to characterize through protocol parsing alone because production vehicles use diverse and proprietary protocol definitions. To address this problem, we propose a protocol-agnostic intrusion detection framework based on multi-scale temporal feature fusion, as illustrated in Figure 3. The framework learns directly from raw CAN traffic and does not require prior knowledge of proprietary protocol details.

To capture these two complementary aspects, the model processes two observation streams in parallel. The first is the intra-ID kinematic sequence, denoted by $F_{intra}$ within a time window $T_{s}$, which forms the kinematic stream and captures the short-term continuity of vehicle dynamics. The second is the inter-ID scheduling sequence, denoted by $F_{inter}$ within a time window $T_{d}$, which forms the scheduling stream and captures timing dependencies among temporally adjacent CAN frames with different identifiers. A multi-scale 1-D CNN is then applied to both streams to extract local temporal features at different receptive fields. To model longer-range dependencies, each stream is further processed by a multi-head self-attention module with positional encoding. Finally, a symmetric cross-attention mechanism is used to fuse the features from the two streams, enabling accurate identification of highly deceptive state-inducing spoofing attacks.

### 4.1. Multi-Scale Temporal Sequence Extraction and Feature Representation

As outlined in Algorithm 1, raw CAN traffic is separated into the intra-ID kinematic sequence $F_{intra}\in R^{T_{s}\times E}$ and the inter-ID scheduling sequence $F_{inter}\in R^{T_{d}\times E}$, where *E* denotes the feature dimension of a single frame. The first stage of the proposed framework uses a 1-D CNN to map these raw sequences into a more discriminative feature space. By convolving along the temporal dimension, the network captures local temporal patterns. The convolution operation is defined as:

(3) $$c_{ij}=\mathrm{ReLU}\left(f_{i}*\omega_{j}\right)+b$$

Here, $f_{i}$ denotes the *i*-th frame vector whose dimension matches the convolution kernel, $\omega_{j}$ is the *j*-th convolution kernel, and *b* is the corresponding bias term. Zero padding is applied throughout the convolutional layers to preserve boundary information.
**Algorithm 1:** Multi-scale temporal sequence extraction scheme
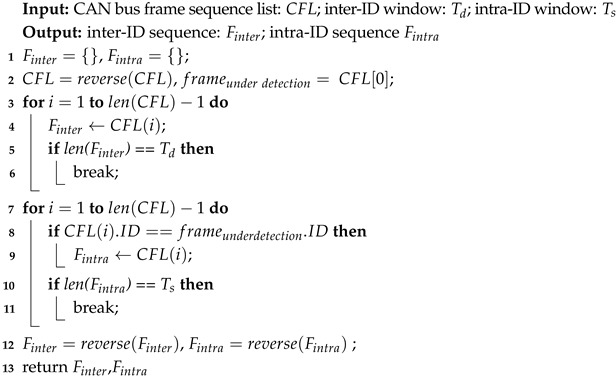


Because the intra-ID sequence $F_{intra}$ reflects the short-term continuity of vehicle dynamics, it exhibits strong local temporal dependencies. For example, frames associated with speed-related identifiers often show stable structural patterns over short time intervals. Therefore, a single convolution kernel of moderate size is sufficient to capture these local temporal characteristics, yielding the intra-ID feature matrix $C_{intra}$:

(4) $$C_{intra}={\left[c_{intra_{ij}}\right]}_{T_{s}\times N}$$

where *N* denotes the number of filters in the convolutional layer.

In contrast, the inter-ID sequence $F_{inter}$ contains both time-triggered periodic patterns and event-triggered variations introduced by scheduling behavior. To capture short-term fluctuations and longer-range scheduling trends at the same time, two convolutional kernels with different sizes are applied in parallel. The smaller kernel focuses on short-range temporal changes, whereas the larger kernel captures broader structural patterns over a longer window. The resulting inter-ID feature matrix $C_{inter}$ is obtained by combining the outputs of the two kernels:

(5) $$C_{inter}={\left[c_{inter_{ij}}\right]}_{T_{d}\times N}^{1}+{\left[c_{inter_{ij}}\right]}_{T_{d}\times N}^{2}$$

It should be noted that insufficient historical frames may occur during vehicle ignition or system startup, especially for low-frequency CAN IDs with long transmission periods. For example, if an ID is transmitted approximately every 1 s, accumulating an intra-ID historical sequence of length $T_{s}$ requires a relatively long initialization time. In contrast, the inter-ID sequence is constructed from temporally adjacent bus frames and usually satisfies the window-length requirement within a much shorter period. Therefore, in practical online deployment, this study adopts a boundary-handling strategy based on buffer initialization and single-stream fallback. The detector maintains a global temporal buffer and an ID-indexed historical buffer. When the inter-ID sequence of the frame under detection already satisfies the length requirement but the intra-ID sequence has not yet been fully initialized, the system falls back to the inter-ID scheduling-stream-only detection mode and outputs a conservative preliminary decision. Once sufficient intra-ID historical frames have been accumulated for that ID, the system automatically switches to the full dual-stream MTFF decision. For offline experiments and performance evaluation, this study only retains samples with complete inter-ID and intra-ID historical windows; warm-up samples with insufficient history are excluded from training, validation, and testing to avoid introducing artificial padding patterns or information leakage caused by borrowing frames across temporal segments.

### 4.2. Hybrid Attention Mechanism-Based Multi-Scale Feature Fusion

Attention mechanisms are widely used in time-series modeling because they can adaptively focus on informative time steps while remaining highly parallelizable. Compared with recurrent architectures such as LSTM and RNN, they are better suited to capturing long-range dependencies. However, self-attention does not preserve the temporal order of sequence elements by itself. Without positional information, a sequence and its reversed version would be treated equivalently. We therefore introduce fixed sinusoidal positional encodings to incorporate temporal order. For an input $C\in R^{T\times N}$, the positional matrix $P={\left[p_{ij}\right]}_{T\times N}$ is defined as

(6) $$p_{i,2j}=sin\left(\frac{i}{1000^{2j/N}}\right)$$

(7) $$p_{i,2j+1}=cos\left(\frac{i}{1000^{2j/N}}\right).$$

The input augmented with positional information is given by

(8) X=C+P.

Multi-head attention extends the basic attention mechanism by using multiple attention heads, allowing the model to capture different temporal relationships in parallel. For the input $X\in R^{T\times N}$, the query, key, and value matrices of each attention head are obtained by linear projections. Specifically, for the *i*-th head, $Q_{i}=XW_{i}^{Q}$, $K_{i}=XW_{i}^{K}$, and $V_{i}=XW_{i}^{V}$, where $W_{i}^{Q},W_{i}^{K}\in R^{N\times d_{k}}$ and $W_{i}^{V}\in R^{N\times d_{v}}$. Thus, $Q_{i},K_{i}\in R^{T\times d_{k}}$ and $V_{i}\in R^{T\times d_{v}}$. The output of a single attention head is computed as

(9) $$\mathrm{Attention}\left(Q_{i},K_{i},V_{i}\right)=\mathrm{softmax}\left(\frac{Q_{i}K_{i}^{T}}{\sqrt{d_{k}}}\right)V_{i}.$$

The output of the multi-head attention mechanism is given by

(10) $$\begin{array}{c} \mathrm{MultiHeadAttention}\left(X\right)\\ =\mathrm{Concat}\left(head_{1},\ldots ,head_{h}\right)W^{O} \end{array}$$

(11) $$head_{i}=\mathrm{Attention}\left(XW_{i}^{Q},XW_{i}^{K},XW_{i}^{V}\right)$$

where *h* is the number of heads and $W^{O}\in R^{hd_{v}\times N}$.

The fusion framework is summarized in Algorithm 2. The intra-ID and inter-ID features extracted by the 1-D CNN are first fed into separate multi-head self-attention modules to model temporal dependencies within each stream. Positional encoding is added at the input stage to preserve temporal order. The resulting features are then fused through a symmetric cross-attention design, in which each stream uses the other as contextual guidance. In this way, the kinematic and scheduling features are integrated while their complementary information is preserved.
**Algorithm 2:** Hybrid-attention-based temporal feature fusion
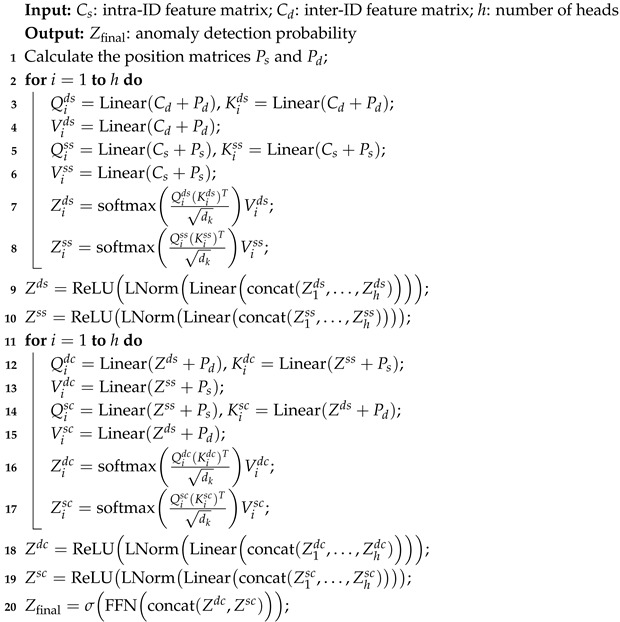


## 5. Experiments and Results

### 5.1. Experiment Setup and Performance Metrics

**Figure 4:**
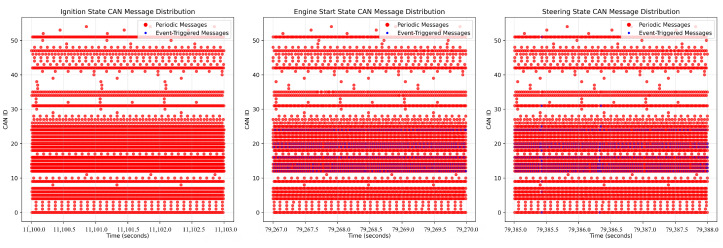
CAN bus frame distribution across ignition, engine running, and steering after engine start in Vehicle A.

**Figure 5:**
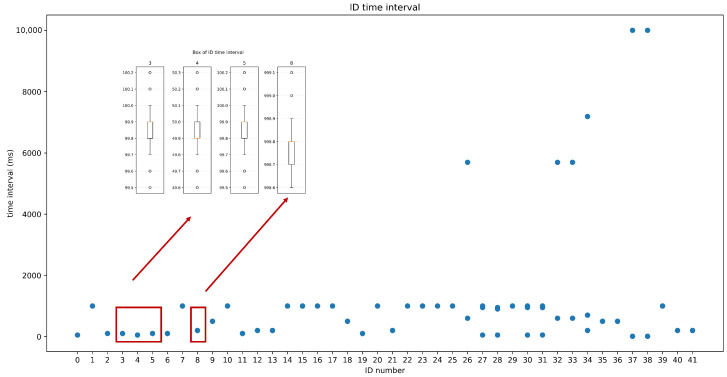
Transmission intervals of the 42 bus IDs in Vehicle B.

To evaluate the proposed model, we collected raw CAN traffic from two production vehicles using hardware transceivers. For Vehicle A, the dataset covers the representative operating states, including ignition, engine running, and steering after engine start. As shown in Figure 4, the traffic evolves from predominantly time-triggered periodic frames during ignition to a more complex mixture with a large proportion of event-triggered aperiodic frames during steering after engine start. For Vehicle B, data collection focuses on steering after engine start, which is the most complex operating condition, in order to further examine the model under highly variable bus behavior. Figure 5 shows the transmission intervals in this condition, where many identifiers exhibit multiple concurrent timing patterns. The blue points represent the observed transmission intervals of the 42 CAN IDs, while the red boxes and arrows link selected IDs to their corresponding boxplots in the inset. For safety and privacy, all original frame identifiers were anonymized.

Most signals exhibit clear periodic behavior, whereas some do not have fixed periods because they are triggered by events. These aperiodic characteristics cannot be reliably captured by ID time-interval statistics alone, which makes attack detection more difficult. In addition, the frame-distribution patterns differ substantially across ignition, engine running, and steering after engine start, further increasing the complexity of detection. The proposed method is designed to address these challenges within a unified framework that covers the representative operating states of the vehicle.

Considering the safety implications of in-vehicle attacks, we reconstructed the collected bus logs in CANoe, Vector’s development environment for real-time ECU bus simulation, analysis, and testing. All attack scenarios were then implemented on this platform. To evaluate the proposed IDS, we adopted four standard metrics: accuracy, precision, recall, and F1-score [29].

The datasets used in this study are summarized in Table 1. For Vehicle A, three representative operating states were considered, including ignition, engine running, and steering after engine start. For each operating state, a continuous CAN trace was extracted from the middle of the raw recording and reported using a 90-s scale to construct the base dataset. For Vehicle B, the same setting was applied to the steering-after-engine-start condition, which was used to further evaluate the proposed method under a more dynamic cross-vehicle scenario.

Based on the above base datasets, four attack scenarios were reconstructed in CANoe, including DoS, fuzzy, replay, and spoofing attacks. For the DoS, fuzzy, and replay attacks, the number of injected attack frames was set to 10% of the corresponding normal frames. For the spoofing attack, three attack ratios, namely 5%, 10%, and 15%, were considered to further evaluate the detection performance under different attack intensities.

During model training and testing, the constructed CAN message sequences were partitioned according to their temporal order. Specifically, for each vehicle, operating state, and attack scenario, CAN messages were first sorted by timestamp, and the training, validation, and testing sets were then constructed from the first 70%, middle 10%, and last 20% of the continuous temporal segments, respectively. The three subsets corresponded to non-overlapping time intervals, and the model input windows were constructed independently within each subset. Samples at the beginning of each subset were removed if their required historical frames were insufficient, rather than borrowing messages from adjacent subsets. The same partitioning strategy was applied to all compared methods to avoid adjacent CAN frames or highly overlapping input windows appearing simultaneously in the training and testing sets, thereby ensuring a fair comparison. The training set was used for model-parameter learning, the validation set for model selection and hyperparameter tuning, and the testing set only for final performance evaluation.

### 5.2. Result

First, we implemented the four attack scenarios described in Section 3 in CANoe. Unless otherwise specified, the term “spoofing attack” in the following experiments refers to the state-inducing spoofing attack defined in Section 3.3. Because payload lengths vary across IDs, all frames were normalized to a fixed length of 64 bits during preprocessing to ensure a consistent input format. It should be noted that the actual payload length of a CAN frame is determined by its DLC and does not necessarily reach 64 bits. For frames with payloads shorter than 8 bytes, the missing byte positions were padded with a sentinel value of 256, which is outside the normal byte range, to form a fixed 64-bit input representation. This preprocessing step is used only to satisfy the fixed-dimensional input requirement of the neural network and does not imply that all real in-vehicle CAN frames carry full 64-bit payloads. We also controlled the ID distribution of attack frames to avoid inflated detection results caused by ID imbalance. For example, if a replay attack involves only a limited set of IDs, the remaining IDs in the test set may be trivially classified as normal. Therefore, except for the DoS attack, the attack frames in the other datasets were generated with a comprehensive and nearly uniform ID distribution. In particular, for replay attacks, we replayed all frame types across 10 attack trials with approximately the same number of replays. It should be emphasized that the nearly uniform ID distribution of attack frames is a controlled experimental design. It is not intended to assume that real attackers necessarily follow a uniform distribution; rather, it is used to reduce simple classification cues caused by attack-ID bias, encouraging the model to rely more on temporal context and payload variations for detection.

**Figure 6:**
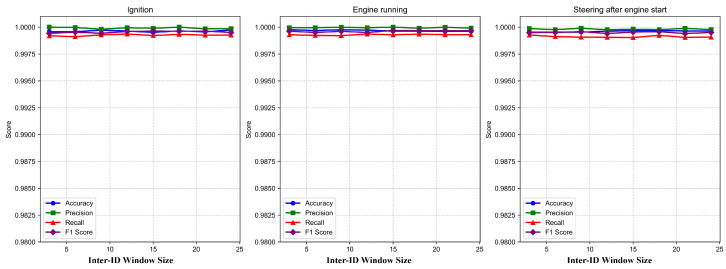
Detection performance of the proposed algorithm against DoS attacks throughout the vehicle multiple operating states of Vehicle A with an intra-ID window of 3.

Figure 6 shows the detection performance of the proposed algorithm against DoS attacks under three operating conditions, with the intra-ID window fixed at 3. Across all operating conditions and inter-ID window sizes, all detection metrics remain above 0.999. This result indicates that the proposed method can detect DoS attacks reliably under different operating conditions. The high performance is mainly due to the clear pattern differences between DoS attack frames and normal CAN traffic.

**Figure 7:**
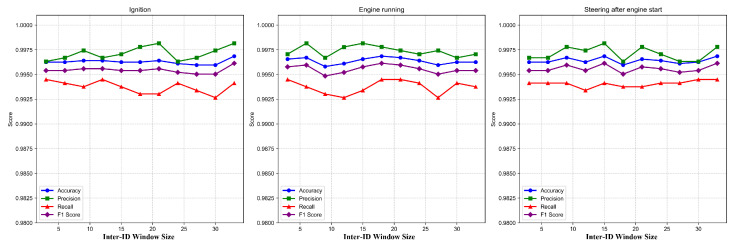
Detection performance of the proposed algorithm against fuzzy attacks throughout the vehicle representative operating states of Vehicle A with an intra-ID window of 3.

Figure 7 shows the detection performance of the proposed algorithm against fuzzy attacks across three operating conditions with the intra-ID window fixed at 3. Compared with DoS attacks, fuzzy attacks are more difficult to detect because the injected frames contain more random identifier and payload patterns. Nevertheless, the proposed method still maintains robust performance under all three operating conditions, namely ignition, engine running, and steering after engine start, with all metrics remaining above 0.992.

**Figure 8:**
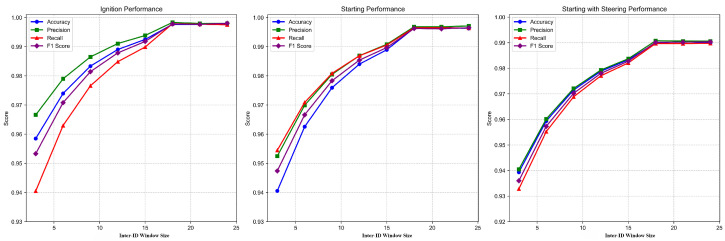
Detection performance of the proposed algorithm against replay attacks throughout the vehicle representative operating states of Vehicle A with an intra-ID window of 3.

Figure 8 shows the detection performance of the proposed algorithm against replay attacks across three operating conditions with the intra-ID window fixed at 3. As the inter-ID window increases from 3 to 18, all detection metrics improve and eventually stabilize above 0.99. Compared with DoS and fuzzy attacks, replay attacks require a larger inter-ID window because replayed frames are structurally similar to legitimate traffic and therefore need more contextual information to be distinguished. By contrast, an intra-ID window of 3 is sufficient, since replay attacks do not require a longer intra-ID temporal context for discrimination.

Spoofing attacks are more challenging than the other three attack types and require richer contextual information for reliable detection. To further evaluate the proposed algorithm under this setting, we consider two attack ratios, 5% and 10%, across three representative vehicle operating states. By varying the intra-ID and inter-ID window sizes, we conduct controlled experiments to examine how window size and attack ratio affect detection performance.

**Figure 9:**
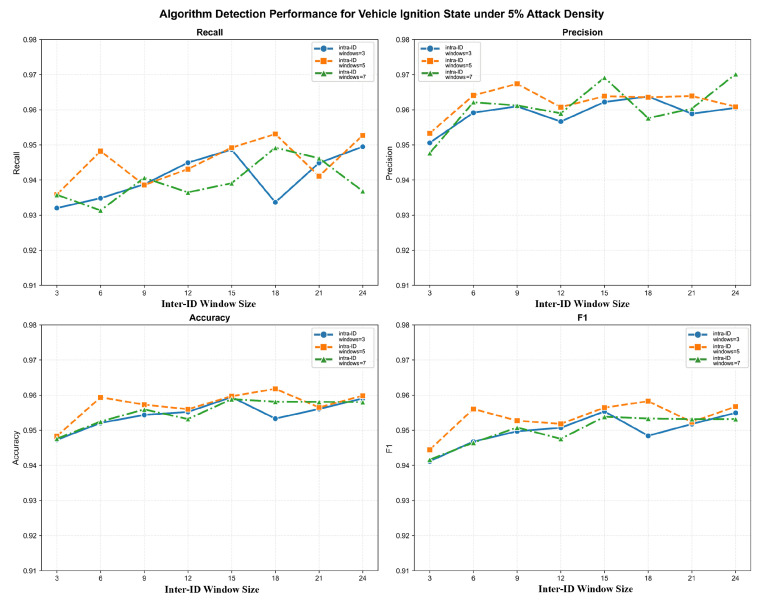
Detection performance against spoofing attacks at a 5% attack ratio during ignition in Vehicle A.

As shown in Figure 9, the proposed algorithm achieves effective detection of spoofing attacks at a 5% attack ratio during ignition in Vehicle A. Detection performance improves as the inter-ID window increases and gradually stabilizes when the window size approaches 18. In addition, increasing the intra-ID window from 3 to 5 further improves the results, indicating that richer temporal context is beneficial for spoofing-attack detection in this setting.

**Figure 10:**
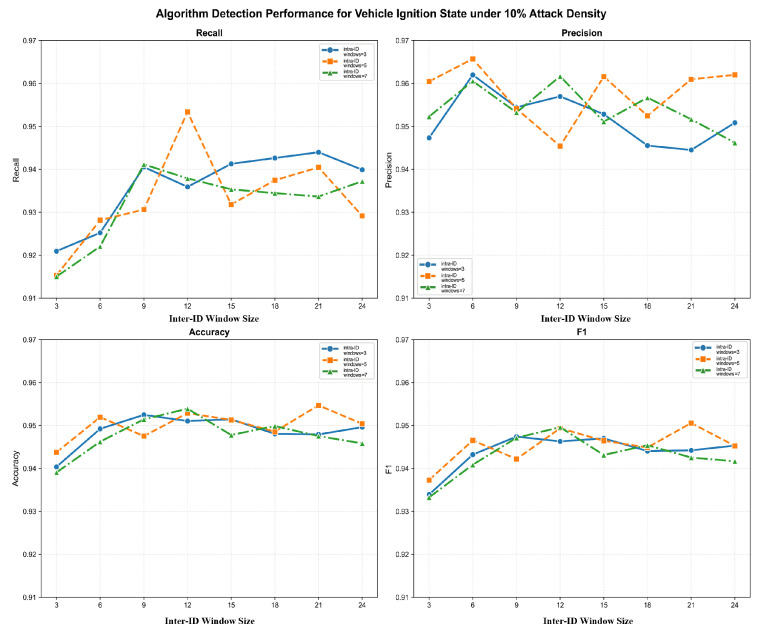
Detection performance against spoofing attacks at a 10% attack ratio during ignition in Vehicle A.

Figure 10 shows that when the attack ratio increases to 10%, the detection performance declines moderately, while the accuracy remains around 0.96. Across both attack ratios, the proposed method still maintains a good balance between recall and precision, indicating stable detection performance under different attack intensities.

**Figure 11:**
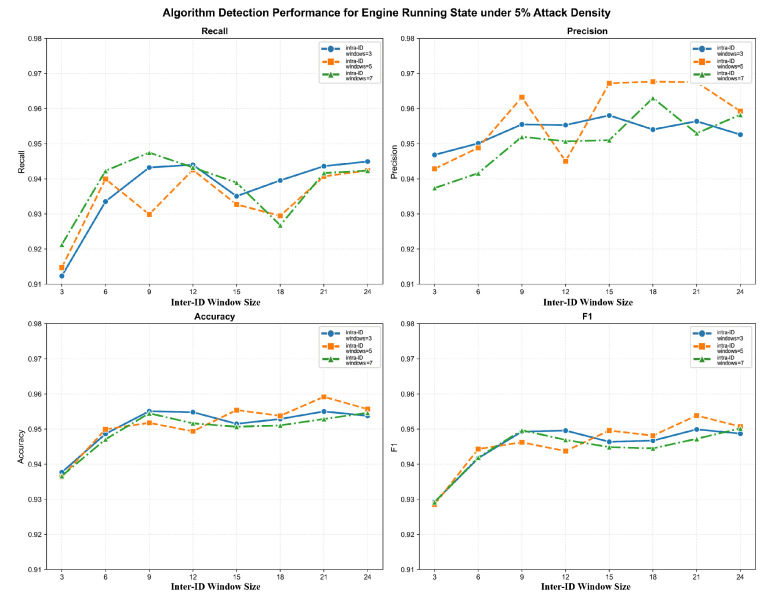
Detection performance against spoofing attacks at a 5% attack ratio during engine running in Vehicle A.

Figure 11 shows the detection performance during engine running at a 5% attack ratio. A trend similar to that observed during ignition can be seen. The best detection performance is achieved when the inter-ID window size is between 18 and 21, indicating that this range provides the most suitable inter-ID context for attack detection under this condition.

**Figure 12:**
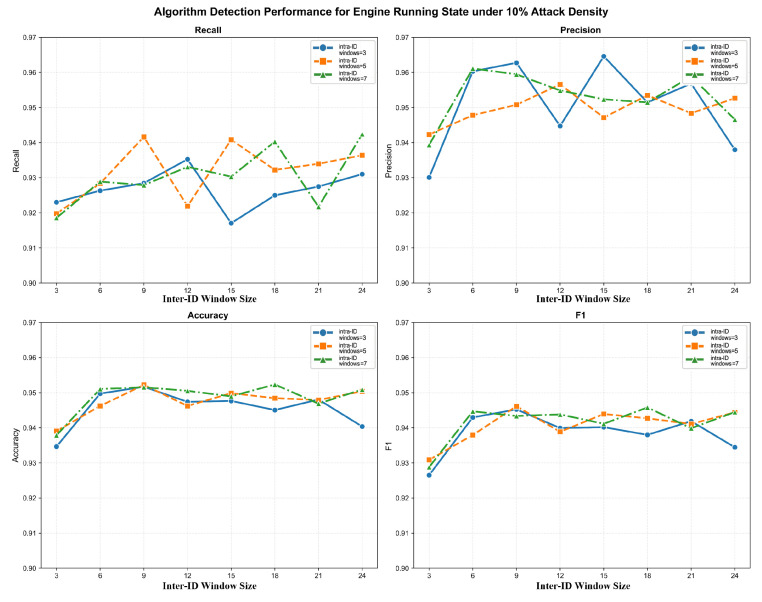
Detection performance against spoofing attacks at a 10% attack ratio during engine running in Vehicle A.

As shown in Figure 12, when the attack ratio increases to 10%, the configurations with intra-ID window sizes of 5 and 7 achieve similar performance, and both clearly outperform the configuration with an intra-ID window of 3. This result indicates that increasing the intra-ID window beyond the minimum value improves detection capability under the engine-running condition.

**Figure 13:**
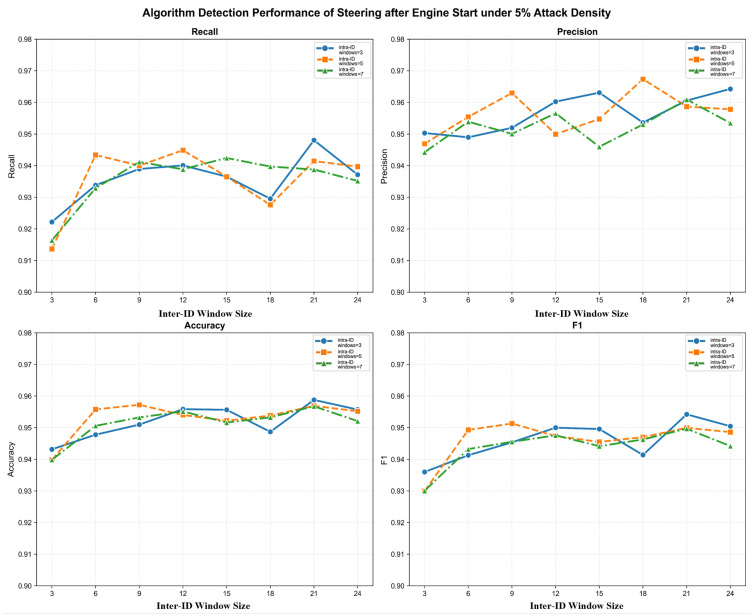
Detection performance against spoofing attacks at a 5% attack ratio during steering after engine start in Vehicle A.

Figure 13 shows the detection performance during steering after engine start at a 5% attack ratio. The best performance is obtained when the inter-ID window size is 21. Meanwhile, the three intra-ID settings of 3, 5, and 7 achieve similar results, indicating that the proposed method remains robust under this attack setting.

**Figure 14:**
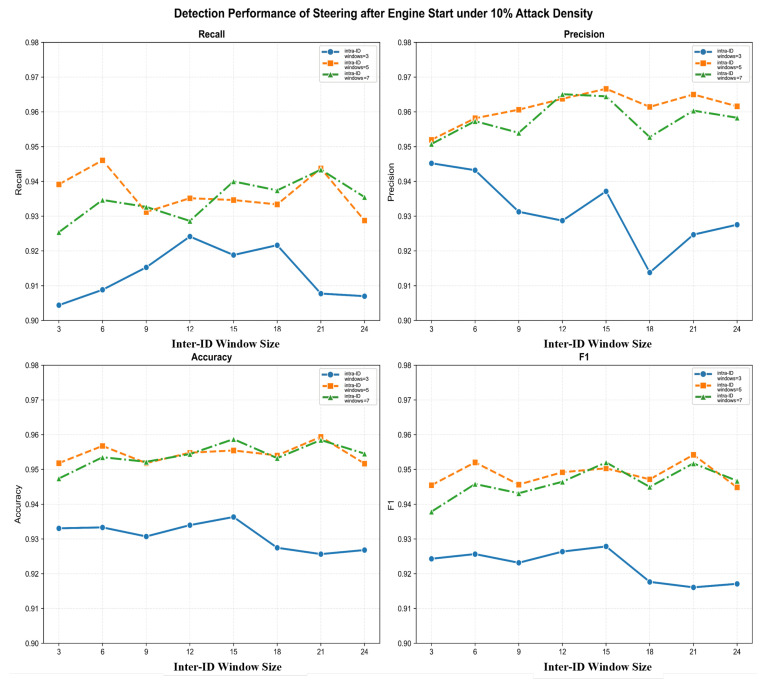
Detection performance against spoofing attacks at a 10% attack ratio during steering after engine start in Vehicle A.

As shown in Figure 14, when the attack ratio increases to 10%, the configurations with intra-ID window sizes of 5 and 7 still achieve stable performance, with all four metrics remaining above 0.95. In contrast, the performance drops to about 0.93 when the intra-ID window is set to 3. This result suggests that under a higher spoofing intensity, a larger intra-ID window provides more discriminative intra-ID temporal context for attack detection.

**Figure 15:**
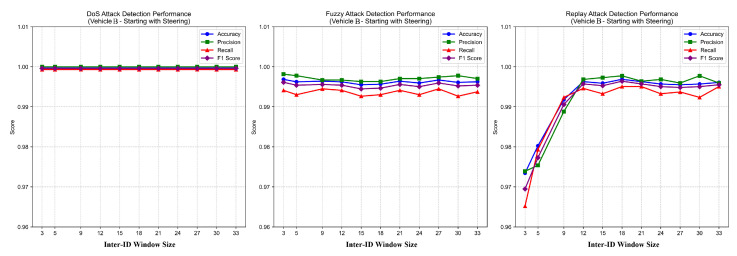
Detection performance against DoS, fuzzy, and replay attacks during steering after engine start in Vehicle B with an intra-ID window of 3.

To further examine the cross-vehicle generalization of the proposed method, we evaluate its detection performance against DoS, fuzzy, and replay attacks during steering after engine start in Vehicle B with the intra-ID window fixed at 3. As shown in Figure 15, the three subplots correspond to the results for these three attack types. For DoS attacks, all four metrics exceed 0.999, indicating that the proposed method can detect this attack type reliably in Vehicle B. For fuzzy attacks, the performance is slightly lower than that for DoS attacks, but all metrics still remain above 0.997. Replay attacks are more difficult because the replayed frames are drawn from legitimate traffic and may appear close to normal frames with the same ID. Accordingly, a larger inter-ID window is required to provide sufficient contextual information. As the inter-ID window increases from 3 to 15, the detection performance improves and gradually stabilizes around 0.99.

**Figure 16:**
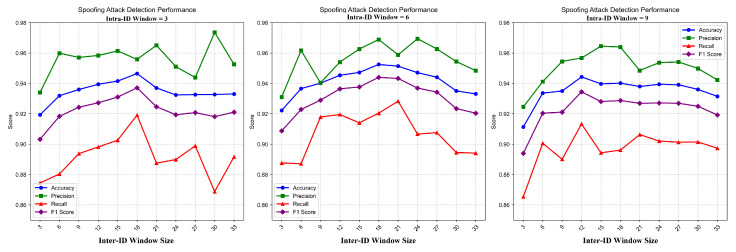
Detection performance against spoofing attacks during steering after engine start in Vehicle B.

Figure 16 analyzes spoofing-attack detection performance during steering after engine start in Vehicle B. The three subplots illustrate the effects of the inter-ID and intra-ID window sizes. In the left panel, increasing the inter-ID window from 3 to 18 improves the detection metrics, indicating that a wider inter-ID context is helpful for spoofing-attack detection. In the middle panel, increasing the intra-ID window from 3 to 6 further improves performance, suggesting that additional intra-ID temporal information is beneficial. In the right panel, however, increasing the intra-ID window from 6 to 9 yields only limited gains. Overall, the best tradeoff is achieved with an intra-ID window of 6 and an inter-ID window between 15 and 18.

Overall, the above results show that the detection performance of MTFF is affected by the settings of the intra-ID window $T_{s}$ and the inter-ID window $T_{d}$, but this effect is closely related to the attack type and vehicle operating condition. For DoS and fuzzy attacks, the injected frames exhibit clear differences from normal traffic in terms of transmission frequency, ID distribution, or payload randomness. Therefore, relatively short temporal windows already provide sufficient discriminative information, and the model is not highly sensitive to the window size. In contrast, replay and spoofing attacks are more similar to normal CAN communication patterns, making them difficult to distinguish from a single frame. These attacks therefore require a longer inter-ID scheduling context and an appropriate intra-ID state-continuity context. The results indicate that increasing $T_{d}$ generally improves the detection of replay and spoofing attacks, but the gain tends to saturate after a moderate window length. Further increasing the window may introduce historical frames that are weakly related to the current frame, especially under complex conditions such as steering after engine start, where event-triggered messages become more frequent and bus scheduling is more variable. Similarly, increasing $T_{s}$ strengthens the modeling of state continuity for the same ID, but an overly long intra-ID window does not necessarily bring additional improvement.

**Figure 17:**
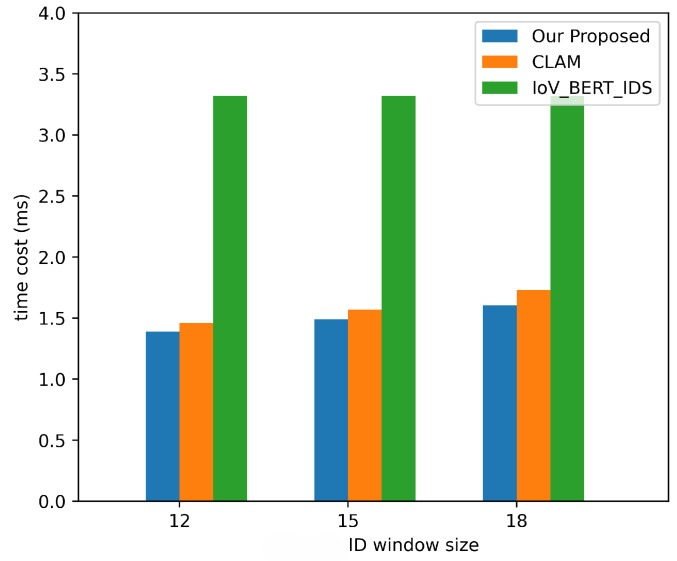
Batch-level detection latency of CLAM, IoV-BERT-IDS, and MTFF.

Specifically, for Vehicle A, DoS, fuzzy, and replay attacks can already be detected effectively when $T_{s}=3$, and the replay-attack performance becomes stable when $T_{d}$ increases to approximately 18. For the more covert spoofing attack, $T_{s}=5$ generally provides more stable performance than $T_{s}=3$, whereas the additional gain from $T_{s}=7$ is limited. Under the most complex steering-after-engine-start condition, $T_{s}=5$ and $T_{d}=18$ provide a reasonable balance between detection performance and contextual length. For Vehicle B, DoS and fuzzy attacks are also insensitive to the window size, while replay-attack detection becomes stable when $T_{d}$ is approximately 15. For spoofing attacks, $T_{s}=6$ clearly outperforms $T_{s}=3$, whereas increasing $T_{s}$ to 9 yields only limited improvement. Therefore, $T_{s}=6$ and $T_{d}=15$–18 are considered a reasonable tradeoff for Vehicle B. Considering that the inference time in Figure 17 increases with the window length, this study treats the window sizes as vehicle-, condition-, and attack-dependent hyperparameters and selects them on the validation set to balance detection performance, model stability, and real-time overhead.

Furthermore, we compare the proposed method with several representative intrusion detection methods, including LSTM [30], BiLSTM [31], BiLSTM with CNN, CLAM [24], and IoV-BERT-IDS [28]. For a fair comparison, all methods were evaluated using the same training, validation, and testing partitions. Each method was independently run with five different random seeds, and the results are reported as the mean ± standard deviation to reflect the performance variation caused by random initialization. In addition, to analyze the contribution of each component in MTFF, we further constructed three ablation variants by removing the intra-ID stream, the CNN feature-extraction module, and the cross-attention fusion module, respectively.

Table 2 reports the comparison and ablation results on Vehicle A under a 10% attack ratio during steering after engine start, where each metric is presented as the mean ± standard deviation over five independent runs. All methods perform well on DoS and fuzzy attacks, but IoV-BERT-IDS and the proposed method achieve better results, especially in identifying attack frames. For DoS attacks, the injected frames use the highest-priority ID and often share relatively fixed payload characteristics with legitimate traffic. When the injection occurs at specific moments, the first few attack frames may therefore closely resemble normal frames with the same ID and be misclassified as normal. A similar effect can also occur in fuzzy attacks, where some injected frames remain close to legitimate traffic. Because IoV-BERT-IDS and the proposed method can model intra-ID temporal features more directly, they are better able to distinguish these hard-to-separate cases.

In the replay attack scenario, the attack frames are direct duplicates of normal frames, which makes detection substantially more difficult than in the DoS and fuzzy scenarios. As shown in Table 2, LSTM-based methods achieve relatively low recall, indicating that a considerable number of replayed frames are missed. This is mainly because replay attacks require the model to capture longer-range temporal dependencies and subtle contextual inconsistencies, which are not modeled effectively by recurrent baselines. Adding CNN-based feature extraction or attention modules on top of LSTM improves the performance to some extent, but the gains remain limited. In contrast, IoV-BERT-IDS and the proposed method are better able to model these contextual patterns and therefore achieve higher detection performance.

In the spoofing attack scenario, adversaries mimic normal traffic patterns in both timing and payload, which makes spoofed frames difficult to distinguish from legitimate traffic. As a result, the recall of all methods is generally lower than their precision. Table 2 shows that, compared with LSTM-based baselines, attention-based methods achieve higher recall while maintaining strong precision. In particular, the proposed MTFF framework, which uses symmetric cross-attention for feature fusion, outperforms IoV-BERT-IDS and the other baselines across all four metrics. These results indicate that MTFF is more effective at reducing both false negatives and false positives in spoofing-attack detection.

The ablation results in Table 2 further show that the performance improvement of MTFF is jointly contributed by the dual-stream representation, local temporal feature extraction, and cross-stream feature fusion. When the intra-ID stream is removed, the performance decreases clearly in the replay and spoofing scenarios, indicating that the historical sequence of the same identifier plays an important role in characterizing vehicle-state continuity. When the CNN module is removed, the model still maintains competitive performance, but the complete MTFF achieves further improvements on fuzzy, replay, and spoofing attacks, suggesting that the multi-scale 1-D CNN helps extract discriminative local temporal patterns. When cross-attention is removed, the performance on replay and spoofing attacks is also lower than that of the complete MTFF, which indicates that cross-stream interaction is useful for exploiting the complementary information between intra-ID state continuity and inter-ID scheduling relationships. In addition, the standard deviations over five independent runs are generally small, suggesting that the observed performance trends are stable and not caused by a single random initialization.

Table 3 presents the comparison and ablation results on Vehicle B under a 10% attack ratio during steering after engine start. Consistent with the results on Vehicle A, the proposed MTFF achieves strong overall performance across all four attack types, which further supports its cross-vehicle generalization capability. In particular, MTFF maintains clear advantages in the replay and spoofing scenarios, where attack frames are more similar to legitimate CAN traffic and require richer temporal context for reliable detection. The ablation results also show a trend consistent with that observed on Vehicle A: removing the intra-ID stream, the CNN module, or cross-attention leads to different degrees of performance degradation, especially for replay and spoofing attacks. This indicates that the dual-stream representation, local temporal feature extraction, and cross-stream fusion contribute consistently across different vehicles. The generally small standard deviations further suggest that the results are stable across different random seeds.

In safety-critical automotive electronic systems, real-time detection is as important as detection accuracy. In vehicular CAN-bus intrusion detection, detection latency measures the time elapsed from the transmission of a malicious CAN frame to its identification by the IDS. According to the system processing pipeline, the total detection latency $T_{\det}$ can be expressed as the sum of the capture latency and the analysis latency:

(12) $$T_{\det}=T_{\mathrm{capture}}+T_{\mathrm{analysis}}$$

Figure 17 compares the inference time required by the proposed method, CLAM, and IoV-BERT-IDS to process one batch, where each batch contains 256 CAN frames. The proposed MTFF and CLAM both exhibit much lower batch-level inference time than IoV-BERT-IDS; in particular, the batch-level inference time of MTFF is approximately 1.4–1.6 ms, corresponding to an average per-frame detection latency of about 0.0055–0.0063 ms. Combined with the preceding detection results, this demonstrates that the proposed method achieves a favorable balance between detection accuracy and real-time performance.

Compared with basic single-stream sequence models or simple rule-based detectors, MTFF introduces multi-scale 1-D CNNs, dual-stream modeling, self-attention, and symmetric cross-attention, and therefore increases model complexity and computational cost. Meanwhile, MTFF uses a compact input representation and only models low-dimensional intra-ID and inter-ID short-window CAN sequences, without relying on large-scale pretrained models or high-dimensional graph inputs, which limits the increase in actual inference overhead. These results indicate that MTFF maintains practical potential for real-time in-vehicle detection while achieving strong detection performance. Deployment on low-end resource-constrained ECUs still requires further validation through model compression, quantization acceleration, and embedded-platform testing.

## 6. Conclusions

This paper proposed a frame-level intrusion-detection framework named MTFF for ICVs across the representative operating states. By decoupling raw CAN traffic into kinematic and scheduling streams, the framework uses multi-scale one-dimensional convolutions and symmetric cross-attention to capture and fuse complementary spatio-temporal dependencies. Hardware-in-the-loop experiments on real-world datasets show that MTFF achieves high detection accuracy and low detection latency against state-inducing spoofing attacks under ignition, engine running, and steering after engine start. Future work will focus on improving the robustness of the model against more sophisticated attacks in production in-vehicle networks.

## Figures and Tables

**Table 1:** Summary of the base CAN datasets.

Vehicle	Operating State	Duration (s)	CAN Frames	CAN IDs
Vehicle A	Ignition	90	156,488	55
Vehicle A	Engine running	90	156,260	55
Vehicle A	Steering after engine start	90	155,669	55
Vehicle B	Steering after engine start	90	136,902	42

**Table 2:** Comparison and ablation results of different methods on Vehicle A under a 10% attack ratio during steering after engine start.

Attack Type	Method	Recall	Precision	Accuracy	F1-Score
DoS	LSTM	0.9957±0.0002	0.9970±0.0002	0.9963±0.0002	0.9963±0.0002
	BiLSTM	0.9958±0.0000	0.9971±0.0000	0.9965±0.0000	0.9965±0.0000
	CNN + BiLSTM	0.9958±0.0000	0.9971±0.0000	0.9965±0.0000	0.9965±0.0000
	CLAM	0.9958±0.0000	0.9971±0.0000	0.9965±0.0000	0.9965±0.0000
	IoV_BERT_IDS	0.9976±0.0000	0.9972±0.0000	0.9975±0.0000	0.9973±0.0000
	MTFF without the intra-ID stream	0.9958±0.0000	0.9971±0.0000	0.9965±0.0000	0.9965±0.0000
	MTFF without the CNN	0.9991±0.0000	0.9995±0.0000	0.9994±0.0000	0.9993±0.0000
	MTFF without cross-attention	0.9991±0.0000	0.9995±0.0000	0.9994±0.0000	0.9993±0.0000
	Proposed MTFF	0.9991±0.0000	0.9995±0.0000	0.9994±0.0000	0.9993±0.0000
Fuzzy	LSTM	0.9592±0.0050	0.9633±0.0024	0.9613±0.0025	0.9612±0.0026
	BiLSTM	0.9732±0.0027	0.9732±0.0038	0.9732±0.0018	0.9732±0.0018
	CNN + BiLSTM	0.9890±0.0020	0.9896±0.0023	0.9893±0.0012	0.9893±0.0012
	CLAM	0.9912±0.0011	0.9910±0.0021	0.9911±0.0014	0.9911±0.0014
	IoV_BERT_IDS	0.9929±0.0016	0.9953±0.0014	0.9942±0.0005	0.9941±0.0006
	MTFF without the intra-ID stream	0.9917±0.0013	0.9909±0.0032	0.9913±0.0015	0.9913±0.0014
	MTFF without the CNN	0.9940±0.0018	0.9956±0.0016	0.9946±0.0006	0.9948±0.0005
	MTFF without cross-attention	0.9954±0.0010	0.9960±0.0017	0.9958±0.0006	0.9957±0.0007
	Proposed MTFF	0.9959±0.0007	0.9972±0.0013	0.9966±0.0005	0.9966±0.0005
Replay	LSTM	0.9270±0.0079	0.9270±0.0055	0.9269±0.0015	0.9269±0.0018
	BiLSTM	0.9373±0.0016	0.9390±0.0017	0.9382±0.0009	0.9382±0.0009
	CNN + BiLSTM	0.9451±0.0011	0.9469±0.0041	0.9460±0.0024	0.9460±0.0023
	CLAM	0.9525±0.0018	0.9602±0.0015	0.9621±0.0012	0.9563±0.0016
	IoV_BERT_IDS	0.9701±0.0035	0.9811±0.0018	0.9757±0.0018	0.9755±0.0018
	MTFF without the intra-ID stream	0.9564±0.0033	0.9598±0.0017	0.9582±0.0021	0.9581±0.0021
	MTFF without the CNN	0.9871±0.0016	0.9899±0.0012	0.9897±0.0011	0.9885±0.0013
	MTFF without cross-attention	0.9801±0.0030	0.9911±0.0015	0.9857±0.0016	0.9856±0.0016
	Proposed MTFF	0.9902±0.0015	0.9928±0.0012	0.9926±0.0010	0.9914±0.0012
Spoofing	LSTM	0.8542±0.0038	0.8937±0.0037	0.8763±0.0030	0.8735±0.0031
	BiLSTM	0.8544±0.0055	0.9026±0.0042	0.8811±0.0039	0.8778±0.0041
	CNN + BiLSTM	0.8888±0.0058	0.9240±0.0048	0.9079±0.0029	0.9061±0.0030
	CLAM	0.9015±0.0040	0.9368±0.0038	0.9204±0.0027	0.9188±0.0027
	IoV_BERT_IDS	0.9096±0.0046	0.9443±0.0040	0.9280±0.0028	0.9266±0.0029
	MTFF without the intra-ID stream	0.9090±0.0031	0.9295±0.0051	0.9201±0.0030	0.9192±0.0029
	MTFF without the CNN	0.9351±0.0070	0.9620±0.0071	0.9490±0.0014	0.9483±0.0014
	MTFF without cross-attention	0.9436±0.0041	0.9615±0.0028	0.9529±0.0006	0.9525±0.0008
	Proposed MTFF	0.9446±0.0027	0.9637±0.0033	0.9545±0.0009	0.9540±0.0008

**Table 3:** Comparison and ablation results of different methods on Vehicle B under a 10% attack ratio during steering after engine start.

Attack Type	Method	Recall	Precision	Accuracy	F1-Score
DoS	LSTM	0.9937±0.0002	0.9949±0.0002	0.9947±0.0002	0.9943±0.0002
	BiLSTM	0.9937±0.0000	0.9950±0.0000	0.9950±0.0000	0.9943±0.0000
	CNN+BiLSTM	0.9937±0.0000	0.9950±0.0000	0.9944±0.0000	0.9943±0.0000
	CLAM	0.9944±0.0000	0.9950±0.0000	0.9945±0.0000	0.9947±0.0000
	IoV_BERT_IDS	0.9955±0.0000	0.9961±0.0000	0.9968±0.0000	0.9958±0.0000
	MTFF without the intra-ID stream	0.9958±0.0000	0.9971±0.0000	0.9965±0.0000	0.9964±0.0000
	MTFF without the CNN	0.9991±0.0000	0.9995±0.0000	0.9994±0.0000	0.9993±0.0000
	MTFF without cross-attention	0.9991±0.0000	0.9995±0.0000	0.9994±0.0000	0.9993±0.0000
	Proposed MTFF	0.9991±0.0000	0.9995±0.0000	0.9994±0.0000	0.9993±0.0000
Fuzzy	LSTM	0.9548±0.0050	0.9607±0.0024	0.9590±0.0025	0.9577±0.0026
	BiLSTM	0.9711±0.0027	0.9712±0.0038	0.9712±0.0018	0.9711±0.0018
	CNN+BiLSTM	0.9869±0.0020	0.9875±0.0023	0.9870±0.0012	0.9872±0.0012
	CLAM	0.9897±0.0011	0.9889±0.0021	0.9887±0.0014	0.9893±0.0014
	IoV_BERT_IDS	0.9910±0.0016	0.9933±0.0014	0.9921±0.0005	0.9921±0.0006
	MTFF without the intra-ID stream	0.9917±0.0013	0.9913±0.0032	0.9915±0.0015	0.9915±0.0014
	MTFF without the CNN	0.9940±0.0018	0.9960±0.0016	0.9948±0.0006	0.9950±0.0005
	MTFF without cross-attention	0.9954±0.0010	0.9964±0.0017	0.9960±0.0006	0.9959±0.0007
	Proposed MTFF	0.9959±0.0007	0.9976±0.0013	0.9968±0.0005	0.9967±0.0005
Replay	LSTM	0.9220±0.0079	0.9250±0.0055	0.9255±0.0015	0.9235±0.0018
	BiLSTM	0.9355±0.0016	0.9380±0.0017	0.9365±0.0009	0.9367±0.0009
	CNN+BiLSTM	0.9420±0.0011	0.9455±0.0041	0.9440±0.0024	0.9437±0.0023
	CLAM	0.9510±0.0018	0.9583±0.0015	0.9602±0.0012	0.9546±0.0016
	IoV_BERT_IDS	0.9825±0.0030	0.9868±0.0018	0.9845±0.0017	0.9846±0.0018
	MTFF without the intra-ID stream	0.9570±0.0033	0.9605±0.0017	0.9590±0.0021	0.9587±0.0021
	MTFF without the CNN	0.9918±0.0015	0.9946±0.0012	0.9935±0.0011	0.9932±0.0013
	MTFF without cross-attention	0.9858±0.0028	0.9948±0.0015	0.9904±0.0015	0.9903±0.0016
	Proposed MTFF	0.9942±0.0014	0.9970±0.0011	0.9960±0.0010	0.9956±0.0011
Spoofing	LSTM	0.8491±0.0038	0.8916±0.0037	0.8742±0.0030	0.8698±0.0031
	BiLSTM	0.8504±0.0055	0.9003±0.0042	0.8790±0.0039	0.8746±0.0041
	CNN+BiLSTM	0.8848±0.0058	0.9221±0.0048	0.9058±0.0029	0.9031±0.0030
	CLAM	0.8974±0.0040	0.9347±0.0038	0.9192±0.0027	0.9157±0.0027
	IoV_BERT_IDS	0.9075±0.0046	0.9422±0.0040	0.9259±0.0028	0.9245±0.0029
	MTFF without the intra-ID stream	0.8991±0.0031	0.9286±0.0051	0.9182±0.0030	0.9136±0.0029
	MTFF without the CNN	0.9252±0.0070	0.9611±0.0071	0.9471±0.0014	0.9428±0.0014
	MTFF without cross-attention	0.9337±0.0041	0.9606±0.0028	0.9510±0.0006	0.9470±0.0008
	Proposed MTFF	0.9347±0.0027	0.9628±0.0033	0.9526±0.0009	0.9485±0.0008

## Data Availability

The data presented in this study are available from the corresponding author upon reasonable request.
